# Thyroid Hormone Upregulates Hypothalamic *kiss2* Gene in the Male Nile Tilapia, *Oreochromis niloticus*

**DOI:** 10.3389/fendo.2013.00184

**Published:** 2013-11-25

**Authors:** Satoshi Ogawa, Kai We Ng, Xiaoyu Xue, Priveena Nair Ramadasan, Mageswary Sivalingam, Shuisheng Li, Berta Levavi-Sivan, Haoran Lin, Xiaochun Liu, Ishwar S. Parhar

**Affiliations:** ^1^Brain Research Institute, School of Medicine and Health Sciences, Monash University Malaysia, Petaling Jaya, Malaysia; ^2^State Key Laboratory of Biocontrol, School of Life Sciences, Institute of Aquatic Economic Animals and the Guangdong Province Key Laboratory for Aquatic Economic Animals, Sun Yat-Sen University, Guangzhou, China; ^3^The Robert H. Smith Faculty of Agriculture, Food and Environment, Department of Animal Sciences, The Hebrew University of Jerusalem, Jerusalem, Israel

**Keywords:** cichlid, *in situ* hybridization, hypothalamus, thyroid receptor, kisspeptin

## Abstract

Kisspeptin has recently been recognized as a critical regulator of reproductive function in vertebrates. During the sexual development, kisspeptin neurons receive sex steroids feedback to trigger gonadotropin-releasing hormone (GnRH) neurons. In teleosts, a positive correlation has been found between the thyroid status and the reproductive status. However, the role of thyroid hormone in the regulation of kisspeptin system remains unknown. We cloned and characterized a gene encoding kisspeptin (*kiss2*) in a cichlid fish, the Nile tilapia (*Oreochromis niloticus*). Expression of *kiss2* mRNA in the brain was analyzed by *in situ* hybridization. The effect of thyroid hormone (triiodothyronine, T_3_) and hypothyroidism with methimazole (MMI) on *kiss2* and the three GnRH types (*gnrh1*, *gnrh2*, and *gnrh3*) mRNA expression was analyzed by real-time PCR. Expression of thyroid hormone receptor mRNAs were analyzed in laser-captured kisspeptin and GnRH neurons by RT-PCR. The *kiss2* mRNA expressing cells were seen in the nucleus of the lateral recess in the hypothalamus. Intraperitoneal administration of T_3_ (5 μg/g body weight) to sexually mature male tilapia significantly increased *kiss2* and *gnrh1* mRNA levels at 24 h post injection (*P* < 0.001), while the treatment with an anti-thyroid, MMI (100 ppm for 6 days) significantly reduced *kiss2* and *gnrh1* mRNA levels (*P* < 0.05). *gnrh2*, *gnrh3*, and thyrotropin-releasing hormone mRNA levels were insensitive to the thyroid hormone manipulations. Furthermore, RT-PCR showed expression of thyroid hormone receptor mRNAs in laser-captured GnRH neurons but not in *kiss2* neurons. This study shows that GnRH1 may be directly regulated through thyroid hormone, while the regulation of Kiss2 by T_3_ is more likely to be indirect.

## Introduction

Kisspeptin, encoded by *Kiss1*/*KISS1* (rodents/human) gene and its cognate receptor, GPR54 (=Kiss-R), have recently been considered the major regulator of reproductive functions, in particular the onset of puberty (1). Administration of kisspeptin stimulates gonadotropin secretion (2), either by its direct action on gonadotrophs (3) or through gonadotropin-releasing hormone (GnRH) neurons (4). Variants of *kiss1* homologous sequences (*kiss1* and *kiss2*) have been identified in several non-mammalian vertebrates including amphibian and teleosts (5, 6). In the teleosts brain, cells expressing *kiss1* mRNA are seen in the ventral habenula and/or the ventral hypothalamus, while those of *kiss2* mRNA are seen in the hypothalamic nuclei and/or the preoptic area depending on the fish species (5, 7). With multiple kisspeptin types, multiple forms of Kiss-R encoding genes (*kissr1* and *kissr2*) have been cloned and characterized in various teleosts (5, 6). Several lines of evidence have demonstrated that Kiss2 is more potent than Kiss1 in the control of reproduction in teleosts (8–11). In the sexually mature zebrafish, *Danio rerio*, administration of Kiss2 peptides significantly increases the gonadotropins β-subunit mRNA levels in the pituitary (9). Similarly in prepubertal European sea bass, *Dicentrarchus labrax*, Kiss2 but not Kiss1 injection increases plasma gonadotropins levels (8). We have previously identified Kiss-R (*kissr2*) in the Nile tilapia, *Oreochromis niloticus* and have shown its expression in GnRH neurons (12). These results suggest the potent role of Kiss2 in the reproductive axis during prepubertal development and sexually mature stages in teleosts. In mammals, kisspeptin neurons transmit gonadal steroid feedback signals to GnRH neurons, especially the positive feedback effect of ovarian estrogen that causes the preovulatory GnRH/luteinizing hormone (LH) surge in female (13). Although the *kiss2* gene is highly conserved in non-mammalian vertebrates, a potent trigger of Kiss2 neural activity has not been identified in teleosts. In the medaka, *Oryzias latipes*, only the hypothalamic *kiss1* but not *kiss2* neurons show prominent estrogen sensitivity in their kisspeptin gene expression (14). Similarly in the goldfish, *Carassius auratus*, the preoptic but not hypothalamic *kiss2* neurons show clear estrogen sensitivity (15). In addition, these estrogen sensitive kisspeptin neuron types in the fish express estrogen receptors (ERα and ERβ) (14, 15). In the juvenile zebrafish, *kiss2* neurons are upregulated by estrogen treatment (16). These observations suggest that the hypothalamic Kiss2 neurons can be regulated by ovarian estrogen in a reproductive stage-dependent manner. However, the concept of an estrogen positive feedback mechanism that initiate the preovulatory GnRH/LH surge is not relevant for males (17). In male aromatase knockout mice, Kiss1 expression in the hypothalamus is not reduced (18). Thus, it is possible that factors other than estrogen play an important role in the regulation of kisspeptin neurons in males (17).

Thyroid hormone is an important regulator of somatic growth, metabolism, brain development, and other vital processes in developing and adult animals (19). Additionally, thyroid hormone also plays an important role in reproductive functions during several physiological conditions (19). In fish, there are numerous studies that examined the effect of hyper- and hypo-thyroidism in sexual development, maturation, and reproductive behavior (20). Direct action of thyroid hormone on GnRH neurons as well as co-expression of thyroid hormone receptors in GnRH neurons has been previously demonstrated (21, 22). In ewe, thyroid hormones are necessary for GnRH and LH pulsatility (23, 24). Although pulsatile secretion of GnRH and kisspeptin are closely interlinked (25), the potential role of thyroid hormone in the regulation of kisspeptin system has never been studied.

In the present study, we cloned *kiss2* cDNA in the Nile tilapia. Gene expression of *kiss2* mRNA in the brain was examined by *in situ* hybridization. Furthermore, to examine the potential role of thyroid hormone in the regulation of the kisspeptin system, the effect of thyroid hormone (triiodothyronine, T_3_) and methimazole (MMI) on *kiss2* and GnRH types (*gnrh1*, *gnrh2*, and *gnrh3*) mRNA expression was analyzed by real-time PCR. MMI inhibits thyroperoxidase, which acts in thyroid hormone synthesis by oxidizing the anion iodide (I^−^) to iodine (I0), facilitating iodine’s addition to tyrosine residues on the hormone precursor thyroglobulin, a necessary step in the synthesis of T_3_ and thyroxine (T_4_). MMI has been shown to reduce plasma thyroid hormone levels and type III deiodinase (D3) activities (hypothyroid condition) in the brain, gill, and liver of tilapia (26). In the present study, to manipulate the plasma thyroid hormone levels in the male tilapia, we applied two different administration methodologies: for hyperthyroid condition, 24 h after intraperitoneal injection of thyroid hormone while for hypothyroid status, fish were immersed in water containing MMI for 6-days. We also measured mRNA expression levels of thyrotropin-releasing hormone (TRH) to validate the effect of thyroid hormone manipulation. Finally, to confirm the potential mechanism of thyroid hormone action on Kiss2 and GnRH neurons, the expression of thyroid hormone receptor (TR) types (*tra1*, *tra2*, and *trb*) mRNA were analyzed in laser-captured *kiss2* and GnRH neurons.

## Materials and Methods

### Animals

Sexually mature male Nile tilapia, *O. niloticus* (standard length: 11.6 ± 0.4 cm, body weight: 52.6 ± 5.0 g) were maintained in freshwater aquaria at 28 ± 0.5°C with a controlled natural photo-regimen (14/10 h, light/dark). They were fed twice daily with commercial tilapia diets (Zeigler, USA). The fish were maintained and used in accordance with the Guidelines of the Animal Ethics Committee of Monash University (Approval Number: SOBSB/2009/58) and Sun Yat-Sen University.

### Molecular cloning of kiss2 in the tilapia

The fish were anesthetized by immersing in a 0.01% solution of tricaine methanesulfonate (MS222; Sigma, St. Louis, MO, USA) and killed by decapitation for sample collection. Total RNA from the tilapia brain (*n* = 1) was prepared using TRIzol reagent (Invitrogen, Carlsbad, CA, USA). One microgram of isolated RNA was used to synthesize the first-strand cDNA using the ReverTra Ace-a first-strand cDNA Synthesis Kit (Toyobo, Osaka, Japan). Partial cDNA fragments were obtained by PCR using degenerate primers or gene-specific primers designed based on the sequences of *kiss* genes of fugu, grouper, medaka, and mackerel (Table 1). Full-length cDNA sequences were obtained by 5′ and 3′ rapid amplification of cDNA ends (RACE) kit (Invitrogen). For all PCR reactions in this study, amplifications were performed with an initial denaturation step at 94°C for 3 min, followed by 40 cycles of 94°C for 15 s, 55-58°C for 15 s, and 72°C for 30 s. The reaction was ended by a further extension of 10 min at 72°C. The amplification products were purified using the E.Z.N.A. Gel Extraction Kit (Omega BioTek, GA, USA) and ligated into the pTZ57R/T vector (Fermentas, MD, USA). Three different individual positive clones were picked to confirm the sequence information using an ABI 3700 sequencer (Applied Biosystems, Foster City, CA, USA). Putative signal peptides and cleavage sites were predicted using SignalP 3.0^1^. Multiple sequence alignments of amino acids were performed with ClustalX (1.81) program. Protein phylogenetic analysis was conducted with MEGA4 using the neighbor-joining method.

**Table 1:** **Primers for tilapia genes used in present study**.

Purpose/genes	Primer direction	5′to 3′sequences
5′RACE	Antisense1	AGGCACCTCCAGTTCTCG
	Antisense2	AGCCATTGTAGCGTTTCC
	Antisense3	CTGCCTCTGGTCCTCGTT
	Antisense4	AGTCGCCTGCTGTTCTCC
3′RACE	Sense1	GAACGAGGACCAGAGGCA
	Sense2	TCTCAGCCTTCGCTTTGG
	Sense3	GGGAAACGCTACAATGGC
	Sense4	CTTGCGAGAACTGGAGGTG
ORF	Sense	TTTGGATCTGGTGCTGAA
	Antisense	GTTTGACTTTCCAAACAAAT
**TISSUE DISTRIBUTION**		
*kiss2*	Sense	GCTTTGGCTGTGGTTTGC
	Antisense	GCCTCTGGTCCTCGTTCT
β*-actin*	Sense	ATGCCTGGCTGGTCCCTCTGTTCT
	Antisense	GGCGGCCAGGTTTGCTATGTA
**REAL-TIME PCR**		
*kiss2*	Sense	TGCACAGAGAACACATGCAA
	Antisense	CTCGCAAGAACAGAGAGAAGG
*gnrh1*	Sense	CTCGCAGGGACGGTGTTT
	Antisense	TCTTCCCTCCTGGGCTCAGT
*gnrh2*	Sense	TGGTCCCATGGTTGGTATCC
	Antisense	CCCTGCTTCACACAGCTTAATCT
*gnrh3*	Sense	TGCTGGCGTTGGTGGTT
	Antisense	CCTCAAGCTCTCCCACACTTCT
*trh*	Sense	GCAGGATGAAGACGGAAGAAAT
	Antisense	GCCGCTTCTCCAAATCATCA
β*-actin*	Sense	CCTGACAGAGCGTGGCTACTC
	Antisense	TCTCTTTGATGTCACGCACGAT
**LOCALIZATION OF TRs**		
*tra1*	Sense	AGTGGAAGCAGAAGCGCAA
	Antisense	TGATGTTGGAGCGACTGGAG
*tra2*	Sense	CCCATCGTCACACCAATGC
	Antisense	TCACAAGGCAGCAGGAATTTG
*trb*	Sense	GAAATTCCTGAGTGCAGCGG
	Antisense	CAGGTGCATTACCCGTGGA
β*-actin*		Same as those used for real-time PCR
*kiss2*		Same as those used for real-time PCR

Primer pairs used for real-time PCR and TRs localization were designed to originate in different exons to exclude false positive bands in case of potential genomic DNA contamination.

Chromosomal location of tilapia *kiss2* gene was identified and its gene synteny with *kiss2* genes in other teleosts (zebrafish, medaka, *O. latipes*, and puffer fish, *Takifugu rubripes*) were examined using the Ensemble Genome Browser^2^.

The tilapia *kiss2* gene promoter sequence was identified *in silico* using the Ensemble Genome Browser. The 2.0-kb sequence upstream of the untranslated region was considered to be the putative promoter. The putative promoter sequence was analyzed for conserved regulatory elements using online bioinformatic tools (TESS^3^; TFSearch^4^; SignalScan^5^).

### Tissue distribution

To determine the tissue distribution of *kiss2* mRNA in the tilapia, sexually mature male and female fish (*n* = 1 each) were anesthetized by immersing in a 0.01% solution of MS222 and killed by decapitation for sample collection. Tissue samples were collected and snap frozen in liquid nitrogen. Total RNA was isolated from the different brain regions (the olfactory bulb, telencephalon, optic tectum thalamus, hypothalamus, cerebellum, and medulla) and peripheral tissues (the pituitary, liver, spleen, intestine, kidney, gill, heart, muscle, testis, and ovary) with TRIzol. One microgram of total RNA from each sample was digested with deoxyribonuclease I (DNase I) and reverse-transcribed into cDNA using the ReverTra Ace-first-strand cDNA Synthesis Kit. PCR was carried out as described above. The PCR products were electrophoresed on 2% agarose gels, stained with ethidium bromide, and visualized by illumination under UV light. All PCR products were confirmed by sequencing.

### *In situ* hybridization

Brains of sexually mature males (*n* = 3) were dissected and fixed in buffered 4% paraformaldehyde for 16 h at 4°C. The brains were then cryoprotected in 20% sucrose and embedded in OCT compound (Sakura Finetechnical, Tokyo, Japan). Consecutive coronal sections (15 μm thick) were cut on a cryostat and thaw-mounted onto 3-aminopropylsilane-coated glass slides. Sense and antisense digoxigenin (DIG)-labeled riboprobes were synthesized from partial sequence of tilapia *kiss2* (266 nt) using MAXIscript (Ambion, Austin, TX, USA) and DIG RNA Labeling Mix (Roche Diagnostics, Mannheim, Germany) according to the manufacturer’s instruction. DIG-*in situ* hybridization was performed as described previously (9). Briefly, sections were subjected to permeabilization with 0.2M HCl for 10 min followed by proteinase K (1 μg/ml) treatment for 15 min, and hybridized with the DIG-labeled riboprobes (50 ng/ml) at 58°C overnight in a humidified chamber. After hybridization, sections were washed and blocked with 2% normal sheep serum. DIG signals were detected with an alkaline phosphatase-conjugated anti-DIG antibody (Roche Diagnostics, diluted 1:500) with 4-nitro blue tetrazolium chloride/5-bromo-4-chloro-3-indolyl-phosphate (Roche Diagnostics).

### Thyroid hormone treatment and induction of hypothyroidism

To induce hyperthyroidism, thyroid hormone (T_3_) was administered as described previously (22). Briefly, anesthetized sexually mature male fish were intraperitoneally (IP) injected with 30 μl of T_3_ (3,3′,5-Triiodo-L-thyronine sodium salt, Sigma, US; dissolved in 100% ethanol and then diluted with sesame oil) at 5 μg/g BW or sesame oil (control) through a 25-gage syringe needle (*n* = 15/group, single injection). The selected dose of T_3_ has been reported to produce plasma T_3_ levels of 4.6 ± 1.2 ng/ml in the male tilapia 24 h after the injection (22), which is within the physiological levels (2 ∼ 5 ng/ml) in the Mozambique tilapia, *O. mossambicus* (27). After the injection, the fish were released into the recovery tank. Twenty-four hours after the injection, the fish were anesthetized and killed by decapitation, and the brain was dissected for RNA isolation.

To induce hypothyroidism, fish were treated in water containing 100 ppm of methimazole (2-Mercapto-1-methylimidazole, MMI, Sigma; dissolved in 100% ethanol and then diluted in water) or in water containing equal volume of 100% ethanol (*n* = 5 ∼ 8/group) for 6-days. The water containing MMI was changed every day, which was required to reduce amount of endogenous T_4_ levels from a euthyroid to hypothyroid state similar to the treatment in tilapia treated with MMI (26). The selected dose of MMI was calculated based on the concentrations that have been applied to the Nile tilapia and the sea bream, *Sparus auratus*, via diet in previous studies (26, 28). After the treatment, the brain tissue was dissected and frozen on dry ice, and stored at −80°C until use for RNA isolation.

### Real-time PCR for kiss2, gnrh1, gnrh2, gnrh3, and trh genes

Total RNA was extracted from the brain with TRIzol (Invitrogen) and 200 ng of total RNA were subjected to cDNA synthesis using High Capacity cDNA Reverse Transcription Kit (Applied Biosystems) in a final volume of 20 μl reaction mixture containing 1× RT buffer, 1× dNTP mix, 1× RT Random Primers, 20U ribonuclease inhibitor, and 10U MultiScribe Reverse Transcriptase according to the manufacturer’s instruction. The cDNA samples were then subjected to real-time PCR for tilapia *kiss2*, *gnrh1*, *gnrh2*, *gnrh3* (GenBank accession numbers for three GnRH types: AB104861, AB101666, and AB104863) and β-actin (*b-actin*) mRNAs with an ABI PRISM 7500 Sequence Detection System (Applied Biosystems). In addition, effect of thyroid hormone manipulation on TRH (also known as thyroliberin: GenBank Accession number, XM_003438996) was also examined. The PCR reaction mixture (10 μl) contained 1× POWER SYBR Green PCR Master Mix (Applied Biosystems), 0.1 μM each forward and reverse primer, and 1 μl of sample cDNA. Nucleotide sequences of real-time PCR primers for tilapia *kiss2*, GnRH types, *trh* and β-actin are presented in Table 1. Reactions were carried out at 50°C for 2 min, 95°C for 10 min, 40 cycles of 95°C for 15 s and 60°C for 1 min followed by a dissociation stage. The cycle threshold (Ct) values of all genes was determined and normalized against β-actin mRNA levels. Data was then analyzed according to relative gene expression by 2^−ΔΔCt^. To check PCR specificity, representative PCR products were electrophoresed on 2% agarose gels, stained with ethidium bromide, and visualized by illumination under UV light. Nucleotide sequences of the PCR products were further confirmed by sequencing. Data are expressed as mean ± SEM and statistical analysis was performed by one-way ANOVA followed by *post hoc* analysis with *t*-test for parametric data or the Mann–Whitney *U* test for non-parametric data with *P* < 0.05 considered significant.

### Expression of TR types in laser-captured kiss2 and GnRH neurons

The expression of TR mRNA types (tra1, tra2 and trb) was examined in laser-captured DIG-labeled *kiss2* neurons and immuno-labeled GnRH types neurons by RT-PCR. The fish were anesthetized by immersing in a 0.01% solution of MS222 and killed by decapitation for sample collection. Brains of sexually mature male fish were processed for DIG-*in situ* hybridization of *kiss2* gene and immunofluorescence labeling of three GnRH neurons types (*n* = 3 for each cell types). DIG-*in situ* hybridization for *kiss2* was performed as described above. For harvesting of GnRH neurons, the brain sections were stained with rabbit anti-tilapia GnRH antibodies against their respective GnRH associated peptide (GAP) sequence (GAP1, #ISP105; GAP2, #ISP205, and GAP3, #ISP305), which were previously generated in our lab. Dilutions (1:1000) were made in an RNase-free phosphate buffer saline (pH 7.0) containing 2% bovine serum albumin and 0.5% triton X-100, and the antiserum was applied to sections mounted on slides for 24 h in a closed moist chamber at 4°C and detected with Alexa Fluor 546-labeled anti-rabbit IgG (1:500 dilution, Invitrogen, Carlsbad, CA, USA). DIG-labeled *kiss2* and immune-fluorescently labeled GnRH neurons were laser-microdissected using an Arcturus XT system (Molecular Devices, Sunnyvale, CA, USA). Each population of the laser-microdissected cells (*kiss2*, ∼200 cells; GnRH1, ∼100 cells; GnRH2, ∼30 cells; GnRH3, ∼30 cells/fish) were placed into sterile 0.2 ml PCR tubes containing 50 μl of lysis solution [1× RT buffer (Applied Biosystems, Foster City, CA, USA), 1% Nonidet P-40, and 0.05 mg/μl proteinase K] and lysed for 1 h at 50°C. After DNase I treatment, total RNA was isolated using TRIzol (Invitrogen) and dissolved in a 10-μl of DEPC-treated water. The total RNAs were subsequently subjected to cDNA synthesis as above. The cDNA samples were then subjected to RT-PCR for tilapia *tra1*, *tra2*, and *trb* (GenBank accession numbers: AF302248, AF302249, and AF302247), β-actin (*b-actin*) and *kiss2* mRNAs. The PCR mixture (20 μl) contained 1× PCR buffer, 160 μM of dNTP mix, 250 nM of forward and reverse gene-specific primers (Table 1), 1U of AmpliTaq Gold DNA polymerase (Applied Biosystems), and one 20th of a single cell’s cDNA solution. Reaction conditions for PCR were 94°C for 10 min, 40 cycles at 94°C for 15 s, 60°C for 15 s, 72°C for 15 s, and 72°C for 7 min. PCR products were electrophoresed on 2% agarose gels, stained with ethidium bromide, and visualized by illumination under UV light.

### Double-immunofluorescence of GnRH1 fibers or GnRHR with kiss2 neurons

To confirm possible associations between GnRH1 and Kiss2 neurons, double-labeling was performed. Kiss2 neurons were detected by fluorescent *in situ* hybridization, while GnRH1 and GnRHR was detected by immunofluorescence. *kiss2* mRNA expressing cells were detected using NEN Fluorescein Tyramide Signal Amplification (TSA™) Plus kit (Perkin Elmer, Wellesley, MA, USA) according to the manufacturer’s instruction. GnRH1-immunoreactive fibers were detected with the anti-tilapia GAP1 antibody (#ISP105, dilution of 1:1000) or anti-tilapia GnRHR [#ISPR3, dilution of 1:500; (29)] with Alexa Fluor 594-labeled anti-rabbit IgG (1:500 dilution, Invitrogen). Separate images were captured by using a microscope (ECLIPS 90i, Nikon Instruments) that was attached to a digital cool CCD camera (DMX1200, Nikon) with appropriate excitation for Fluorescein and Alexa Fluor 594, and a computer software (NIS Elements D3.0, Nikon) was used to superimpose the two images. The red channel was then converted to magenta, and brightness and contrast adjustments were made in Adobe Photoshop CS2 (Adobe Systems, San Jose, CA, USA).

## Results

### Cloning and sequence analysis of tilapia kiss2 cDNA

**Figure 1:**
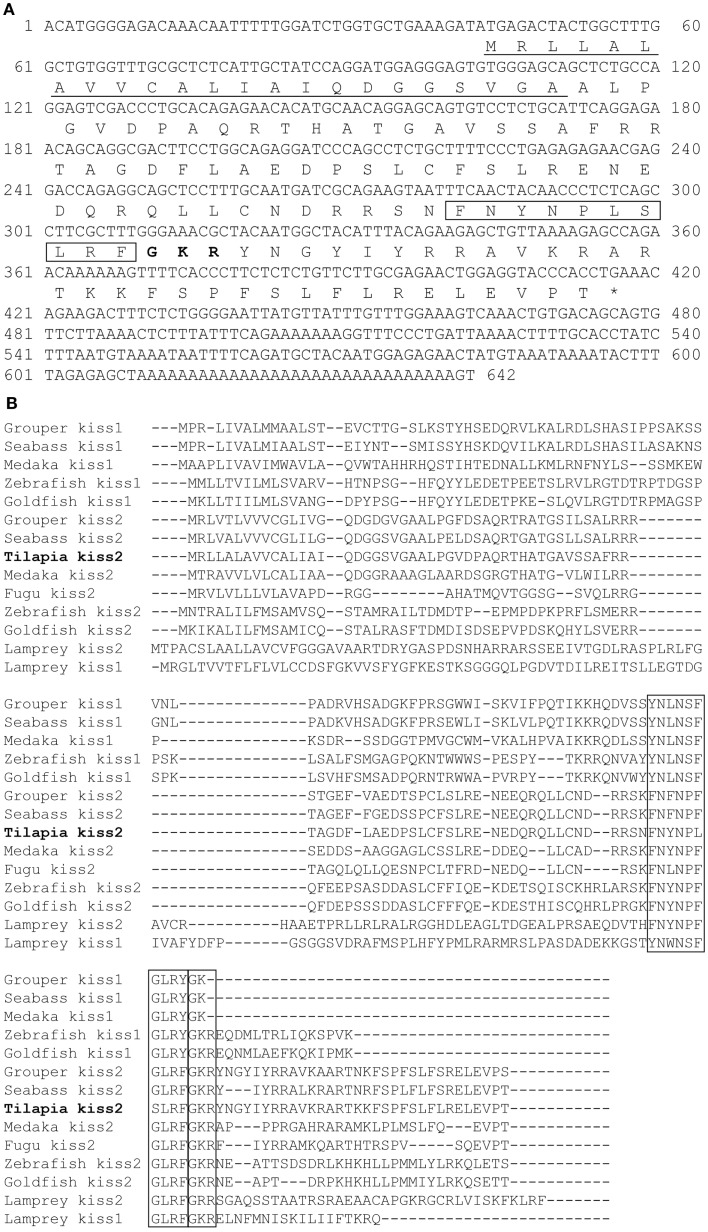
**cDNA and deduced amino acid sequence of tilapia *kiss2***. **(A)** Nucleotide and deduced amino acid sequence of tilapia *kiss2*. Putative signal peptide (underlined) were predicted using SignalP 3.0 (http://www.cbs.dtu.dk/services/SignalP/). Putative core peptide is boxed. Potential cleavage amidation site (GKR) is bolded. The stop codon is denoted by an asterisk. **(B)** Comparison of amino acid sequences of kisspeptin precursors from different species. The mature peptides and potential cleavage amidation site (GKR/GK/GKK) are boxed. Sequences were aligned by the ClustalW program. Gaps (indicated by hyphens) are introduced in some sequences to maximize alignment.

**Figure 2:**
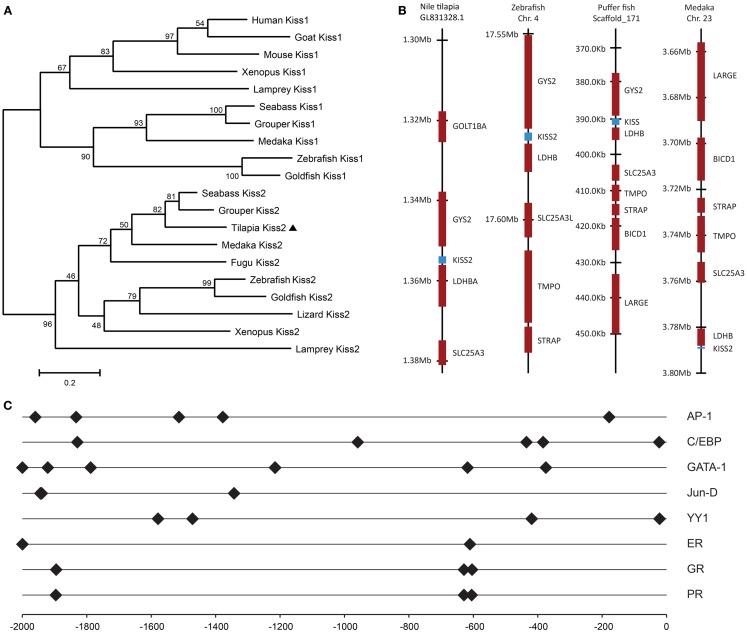
**Genomic analysis of tilapia *kiss2* gene**. **(A)** Phylogenetic analysis of KISS precursors in vertebrates. The phylogenetic tree was constructed by MEGA 4.0.2 using the neighbor-joining method with 1000 bootstrap replicates. The number shown at each branch indicates the bootstrap value (%). GenBank accession numbers for KISS: human KISS1 (NP_002247.3); Mouse KISS1 (AAI17047.1); Goat KISS1 (ACI96030.1); Clawed frog Kiss1 (NM_001170453.1); Clawed frog Kiss2 (NM_001162860.1); Zebrafish Kiss1 (NP_001106961.1); Zebrafish Kiss2 (NP_001136057.1); Grouper Kiss1 (ADF59544.1); Grouper Kiss2 (ADF59545.1); Medaka Kiss2 (NP_001153913.1); Fugu Kiss2 (BAJ15497.1); Sea bass Kiss1 (ACM07422.1); Sea bass Kiss2 (ACM07423.1). Sequences predicted from Ensembl: sea lamprey Kiss2 (Contig Contig37453.1 at location 1700–6241); Lizard Kiss2 (on scaffold_15 at location 4,601,534–4,601,935); Sea lamprey Kiss1 sequence were previously predicted by van Aerle et al. (30); Goldfish Kiss2 were obtained by Li et al. (31). **(B)** Chromosomal locations *kiss2* (blue box) in various teleosts species. **(C)** Putative transcription factor binding sites (closed diamond) on the promoter region of tilapia *kiss2* gene. The numbers −2000 to 0 represent distance in bp from the putative transcriptional initiation site.

A full-length cDNA encoding the *kiss2* precursor was isolated from the tilapia, and the cDNA sequence has been deposited in the GenBank (accession number JN565693). The cDNA encoding tilapia *kiss2* is 633 base pairs (bp), containing an open reading frame of 375 bp, 35 bp of 5′-UTR, and 223 bp of 3′-UTR. The Kiss2 precursor protein has 124 amino acids (aa), with an N-terminal putative signal peptide sequence of 23 aa and a cleavage site (GKR) (Figure 1A). Sequence comparison of the deduced protein sequences showed that the tilapia and other vertebrate Kiss precursor proteins are poorly conserved (Figure 1B). However, the mature peptide (Kiss2–10) of tilapia and other species exhibit relatively conserved, differing by two amino acid at the position 6 and 7 (phenylalanine to leucine and glycine to serine) (Figure 1B). Phylogenetic analysis showed that kisspeptin deduced protein sequences are clustered into two separate clades: Kiss1 and Kiss2. The tilapia Kiss2 is clustered with the Kiss2 clade and shares the highest similarity with sea bass and grouper Kiss2 (Figure 2A).

### Gene synteny analysis

Tilapia Kiss2 encoding sequence was found in the chromosome, scaffold GL831328.1 (location 1,353,904–1,355,714). Chromosome synteny analysis revealed that the neighborhood genes around the tilapia *kiss2* including *ldhba* and *slc25a3* are conserved in other fish Kiss2 genes (Figure 2B). Some of gene loci nearby the tilapia Kiss2 including *goltlba* and *gys2* were also found on human chromosome 12 and mouse chromosome 6 as reported previously (9).

### Putative transcription factor binding sites on the promoter region of tilapia *kiss*2 gene

*In silico* analysis of putative transcription binding sites showed the presence of binding sites for several transcription factors such as AP-1, CEBP, GATA-1, Jun-D, YY1, ER, GR, and PR on the putative promoter region of tilapia *kiss2* gene (Figure 2C).

### Tissue distributions and brain localization of tilapia kiss2 mRNA

**Figure 3:**
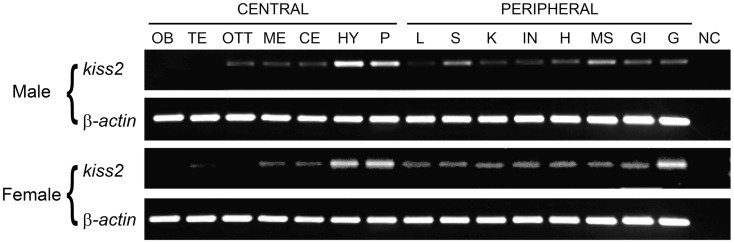
**RT-PCR analysis of tissue expression patterns of *kiss2* in male and female tilapia**. Amplification of β-actin was used as the house-keeping gene control. OB, olfactory bulb; TE, telencephalon; OTT, optic tectum thalamus; ME, medulla; CE, cerebellum; HY, hypothalamus; P, pituitary; L, liver; S, spleen; K, kidney; IN, intestine; H, heart; MS, muscle; GI, gill; G, gonad; NC, negative control.

RT-PCR analysis was performed to examine the tissue distribution patterns of the tilapia *kiss2* gene expression. In the brain, the tilapia *kiss2* mRNA was highly expressed in the hypothalamus and the pituitary in males and females (Figure 3). In peripheral tissues, there were sexual differences in the distribution patterns. In males, the tilapia *kiss2* mRNA was expressed in the spleen, medulla, gills, and testis, whereas in females, the tilapia *kiss2* mRNA was expressed in the spleen, kidney, intestine, heart, medulla, gills, and ovary (Figure 3).

**Figure 4:**
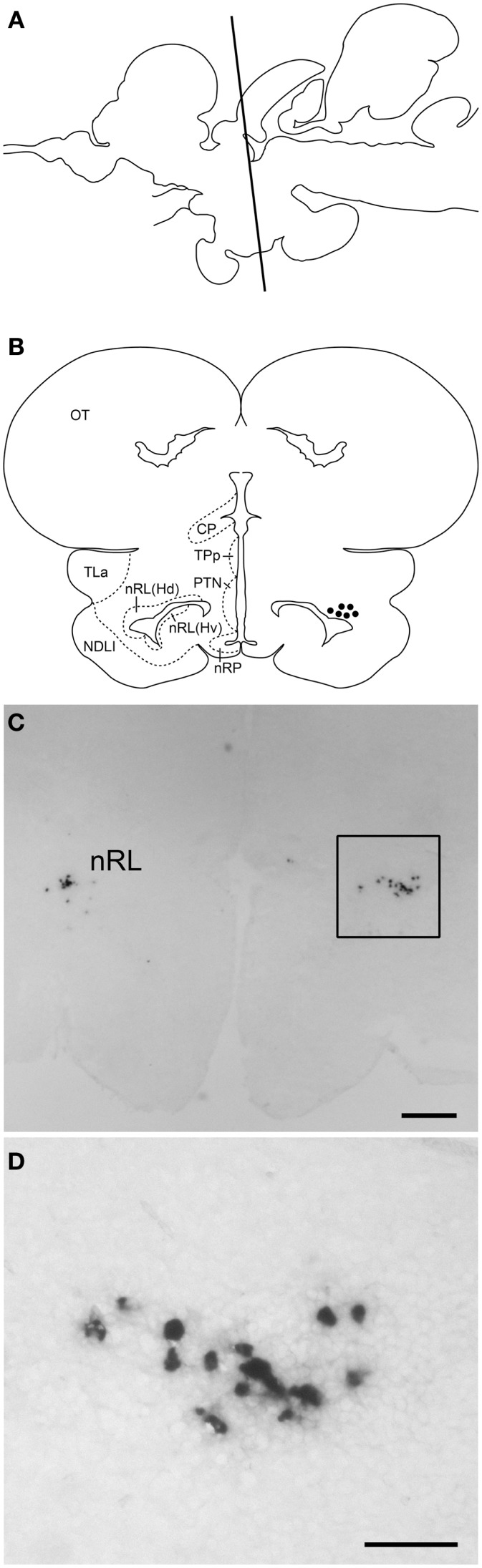
***kiss2* mRNA containing cells in the brain of male tilapia**. **(A)** Schematic drawing of a lateral view of the brain indicating levels of the corresponding transverse section. **(B)** Schematic drawing of transverse section of *kiss2* cells (closed circles). **(C,D)** Photomicrographs of DIG-labeled *kiss2* cells in the nucleus of lateral recesses (nRL) at **(C)** Low- and **(D)** high-magnification. CP, central posterior thalamic nucleus; Hd, dorsal zone of periventricular hypothalamus; Hv, ventral zone of periventricular hypothalamus; NDLI, diffuse nucleus of the inferior lobe; nRP, nucleus of posterior recess; OT, optic tectum; PTN, posterior tuberal nucleus; TLa, torus lateralis; TPp, periventricular nucleus of posterior tuberculum. Scale bars: (**C)** 200 μm; **(D)** 50 μm.

Digoxigenin-*in situ* hybridization showed tilapia *kiss2* mRNA containing cells in the nucleus of the lateral recess [nRL, also been referred to as the dorsal zone of the periventricular hypothalamus (32)] in the brain (Figure 4). No DIG-labeled cells were detected in the brain using sense riboprobes (data not shown).

### Effect of thyroid hormone (T_3_) and hypothyroidism on kiss2, GnRH types and TRH gene expression

**Figure 5:**
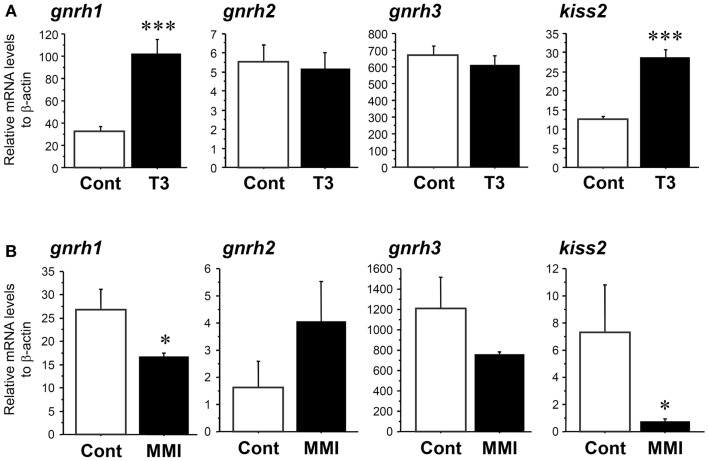
**Effect of thyroid hormone manipulation in *kiss2*, *gnrh1*, *gnrh2*, and *gnrh3* mRNA levels in the male tilapia**. **(A)** Thyroid hormone (T_3_, 5 μg/g body weight) injection significantly increased *kiss2* and *gnrh1* mRNA levels (*n* = 15). **(B)** Under hypothyroidism with methimazole (MMI, 100 ppm for 6 days), mRNA levels of *kiss2* and *gnrh1* were significantly decreased. There were no effects of manipulation of thyroid hormone on *gnrh2* and *gnrh3* mRNA levels (*n* = 5–8). The relative abundances of the mRNA were normalized to the amount of β-actin using the comparative threshold cycle method. *P < 0.05; ***P < 0.001 vs. controls.

Real-time PCR showed that administration of T_3_ significantly increased the amount of *kiss2* (∼2.3-fold, *P* < 0.001) and *gnrh1* (∼3.2-fold, *P* < 0.001) mRNA levels 24 h post administration when compared with control fish (Figure 5A). There was no effect of T_3_ treatment on *gnrh2* (*P* = 0.86) and *gnrh3* (*P* = 0.47) mRNA levels (Figure 5A).

In the fish treated with MMI, the amount of *kiss2* (∼0.1-fold, *P* < 0.05) and *gnrh1* (∼0.6-fold, *P* < 0.05) mRNA levels were significantly decreased compared with control fish (Figure 5B). There was no effect of MMI treatment on *gnrh2* (*P* = 0.08) and *gnrh3* (*P* = 0.14) mRNA levels (Figure 5B).

**Figure 6:**
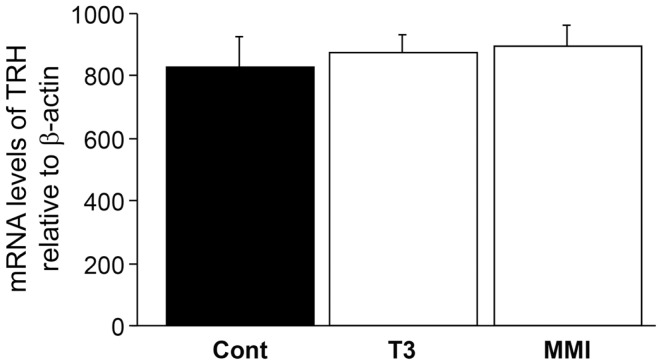
**Effect of thyroid hormone manipulation on thyrotropin- releasing hormone (TRH) mRNA levels**. There was no significant effect of thyroid hormone (T3) and MMI on TRH mRNA levels in the male tilapia.

There was no significant effect of thyroid hormone injection and MMI treatment on TRH mRNA levels in the brain (Figure 6), indicating the absence of endogenous thyroid hormone feedback effect on *kiss2* mRNA levels.

### Expression of TR types in laser-captured kiss2 and GnRH neuron types

**Figure 7:**
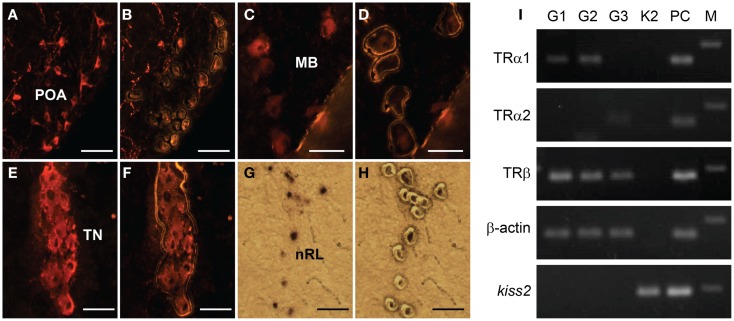
**Expression of thyroid hormone receptor (TR) types mRNA in laser-captured GnRH1, GnRH2, GnRH3, and *kiss2* neurons**. **(A–H)** Photomicrographs of GnRH1 **(A,B)**, GnRH2 **(C,D)**, GnRH3- immunoreactive **(E,F)**, and DIG-labeled *kiss2* **(G,H)** neurons before **(A,C,E,G)**, and after **(B,D,F,H)** laser-capture microdissection. Scale bars, 50 μm. POA, preoptic area; MB, midbrain; TN, terminal nerve; nRL, the nucleus of lateral recesses. **(I)** RT-PCR of the *tra1*, *tra2*, *trb*, β-actin, and *kiss2* genes in the laser-captured immune-fluorescently labeled three GnRH types neurons (G1, G2 and G3) and DIG-labeled *kiss2* neurons (K2). PC, Whole-brain cDNA as a positive control; M, 100-bp DNA ladder.

RT-PCR showed no expression of TR types (*tra1*, *tra2*, and *trb*) mRNA in laser-captured *kiss2* cells (Figure 7). In GnRH neuron types, expression of *tra1* mRNA was found in GnRH1 and GnRH2 neurons, *tra2* mRNA was found in GnRH3 neurons, and *trb* mRNA was found in GnRH1, GnRH2, and GnRH3 neurons (Figure 7).

### Possible neuronal associations between GnRH1 and kiss2 neurons

**Figure 8:**
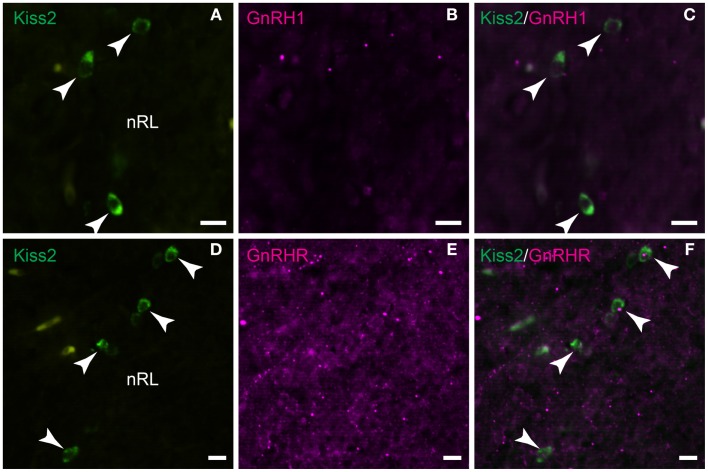
**Double-label immunofluorescence photomicrographs of tilapia *kiss2* and GnRH1 or GnRHR in the brain of male tilapia**. **(A–C)** There was no GnRH1 (GAP1)-immunoreactive fibers (magenta) nearby *kiss2* mRNA containing cells (green, arrow heads) in the nucleus of lateral recesses (nRL). **(D–F)** There was no co-expression of GnRHR immunoreactivity (magenta) in *kiss2* mRNA containing cells (green, arrow heads). Scale bars, 10 μm.

Double-immunofluorescence showed neither close association of GnRH1-immunoreactive fibers with *kiss2* neurons (Figures 8A–C) nor co-expression of GnRHR-immunoreactivity in *kiss2* neurons (Figures 8D–F).

## Discussion

### Kiss2 gene in the tilapia

The core sequence of tilapia Kiss2 showed high similarities with non-mammalian Kiss2 peptides sharing the F–F form. However, there are the two major amino acid substitutions at positions 6 and 7 (Leu-Ser instead of Phe-Gly) in the core sequence of tilapia Kiss2 decapeptides. As a result, the carboxyl half of core peptide of tilapia Kiss2 (position 6 to 10) is LSLRF, while those of all other Kiss2 identified thus far are FGLRF with complete conservation from lamprey, elephant shark through platypus that have been appeared to possess Kiss2 (7). These two amino acids substitution could be important for binding affinity to Kiss-R and calcium release activity (33). Comparison of genomic sequences showed conserved synteny between the tilapia, zebrafish, puffer fish and medaka, suggesting tilapia Kiss2 gene is ortholog. So far no *kiss1* and *kissr1* homologous sequences have been reported in the tilapia, similar to those fish that possess only one *kiss*-*kissr* gene (5, 11). Nevertheless, the presence of *kiss1* in the tilapia remains to be examined.

The expression pattern of *kiss2* mRNA in various tissues in the tilapia is similar to that in other fish species (5, 7). In the brain, *kiss2* mRNA containing cells were seen only in the nRL. However, no *kiss2* cells were seen in other brain region such as the posterior tuberal nucleus or the preoptic area where *kiss2* cells exist in the medaka, zebrafish, goldfish, red seabream (*Pagrus major*) and European sea bass (*D. labrax*) (9, 15, 34–36), which could be due to species difference or because of its low expression levels in the preoptic region. Expression of kisspeptin genes in the pituitary have been reported in several species including mammals and fish (7, 36, 37). The presence of *kiss2* mRNA in the pituitary of tilapia indicates the possibility of *kiss2* mRNA being transported to the nerve terminals as seen in some neuron types (38), or being expressed locally in the pituitary similar to *kiss1* mRNA in the European sea bass and Kiss2-immunoreactive cells in the zebrafish (16, 36). The target site of tilapia Kiss2 neurons is still unknown due to the lack of specific antibody. A recent study in the zebrafish has demonstrated projections of Kiss2-immunoreactive fibers throughout the brain and their close association with GnRH3 (hypophysiotropic GnRH type in the zebrafish) neurons in the preoptic area (16), which suggest the primary role of Kiss2 neurons in gonadotropin secretion possibly through the stimulation of GnRH. It has been shown that electrical stimulation of the nRL in teleosts elicits feeding, gravel picking, and generally aggressive behaviors in cichlids (39). A recent study has shown significant increase in *kiss2* mRNA levels in the hypothalamus during fasting conditions in the Senegalese sole (*Solea senegalensis*) (40). These observations suggest the potential role of Kiss2 in homeostatic regulation as well as ingestive and sexual behaviors as suggested in mammals (41).

The predominant expression of *kiss2* in the brain, testis, and ovary suggests its role in reproductive functions. Specific localization of *kiss2* mRNA in the gonadal tissues has not been studied in teleost, but in the cyclic human and marmoset ovaries, kisspeptin-immunoreactive signals have been located in the theca layer of growing follicles, corpora lutea, interstitial gland, and ovarian surface epithelium (42). Similarly in teleosts, Kiss2 peptides could be locally synthesized in gonadal tissues and could regulate gonadal maturation.

### Effect of thyroid hormone on reproductive neuroendocrine system

The manipulation of thyroid hormone levels significantly altered *kiss2* mRNA levels along with *gnrh1* mRNA levels in the brain of male tilapia. Furthermore, there was no effect of thyroid hormone manipulation on *gnrh2* and *gnrh3* mRNA levels. These results indicate that thyroid hormone may act on kisspeptin-GnRH1 system which plays an important role in the reproductive neuroendocrine axis in the tilapia. A recent study in primates has proposed kisspeptin neurons as candidate action target of thyroid hormone (43). The regulation of GnRH neurons by kisspeptin is critical for the onset of puberty. During the prepubertal stage, sex steroids as well as thyroid hormone are involved in the development of the sexually mature brain. In the quail, thyroid hormone has been reported to cause seasonal change in the morphology of GnRH nerve terminals at the median eminence (44). In monkeys, hypothyroid condition with MMI treatment during the juvenile stage delays the pubertal rise in LH secretion and only 50% of the hypothyroid animals exhibit reactivation of GnRH pulse generator activity (45). In teleosts, there are limited studies that examined the role of thyroid hormone in the regulation of GnRH neurons. In the larval tilapia, the concentration of thyroid hormone levels in the whole-body peak around day 25 after hatching (46), which correspond with the period when GnRH1-immunoreactive cells are morphologically detectable in the preoptic area (47). In the zebrafish, the timing of first appearance of preoptic GnRH3 neurons and that of the increase in *gnrh3* gene expression coincides with the second peak of *kiss2* gene expression (9). However, these studies only support the potential organizational effect of thyroid hormone on the reproductive axis in juvenile or seasonal breeding animals, which is not the case for the present study that demonstrates the activational effect of thyroid hormone on kisspeptin-GnRH axis in the sexually mature fish. Nevertheless, even in non-seasonal breeding animals, thyroid hormone levels are influenced by various factors such as diurnal rhythm (photoperiod), metabolism, and stress, that have direct or indirect impact on the kisspeptin system (48, 49), which may alter the release of GnRH.

We previously found the significant effect of thyroid hormone on *gnrh1* mRNA levels in sexually mature but not in immature tilapia (22). This result suggests that in sexually mature fish, GnRH1 neurons may acquire sensitivity to thyroid hormone due to the presence of TR, which might be absent in sexually immature fish. Similarly in male monkeys, hypothyroidism fails to prevent the arrest of GnRH pulse generator activity during the infant-juvenile transition (43). Therefore, the action of thyroid hormone on kisspeptin-GnRH neurons could be regulated in a reproductive stage-dependent manner.

### Effect of thyroid hormone on GnRH neurons: Direct and indirect pathways

The role of thyroid hormone in reproductive functions is important during developmental as well as in the adult stages. In rats, irregular estrous cycle, failure of LH surge, impairment in male sexual behavior, and reduction of GnRH biosynthesis has been shown when hypothyroidism was induced during their adult stage (50–52). A recent study in the rat has shown the presence of type II deiodinase in GnRH neuronal axons in the median eminence as well as in GT1-7 cells (53), indicating the possible synthesis of thyroid hormone within GnRH neurons and possible direct action of thyroid hormone on GnRH neurons. In the present study, we found the expression of TR mRNA types in GnRH1 neurons, which also has been reported in the sheep and hamsters (21). The promoter region of rat GnRH gene contains motifs resembling ER/TR response elements (54). Furthermore, the rat GnRH promoter contains a retinoic acid response element (54), which can interact with TRs alone or with TR/retinoic acid receptor heterodimers (21). Therefore, thyroid hormone can directly act on GnRH1 neurons to regulate the synthesis of GnRH peptides.

In the reproductive axis, pulse, and surge pattern of GnRH secretion are critical. Recent studies in mammals have suggested that kisspeptin neurons in the arcuate nucleus (Arc) are responsible for the pulsatile release of GnRH (55). In ewes, thyroid hormones are required for steroid-independent seasonal LH pulse frequency (24), in which LH pulse frequency and amplitude alters in the absence of estradiol (56). This could be mediated through TR localized in the Arc (57) that contains kisspeptin neurons. Although kisspeptin has been considered a major regulator of GnRH neurons, a morphological study in the rhesus monkey has shown occasional contacts between GnRH axons and kisspeptin neurons in the Arc, indicating the possibility that GnRH could exert control over kisspeptin neuronal activity (58). However, in this study, we failed to observe any GnRH1 fibers or GnRHR in Kiss2 neurons. Therefore, it is possible that the thyroid hormone indirectly regulates Kiss2 neurons via unidentified neuronal population expressing conventional TR.

In the present study, we failed to observe the expression of TR mRNA types in Kiss2 population, which could be due to low expression levels of TR genes in *kiss2* neurons. The absence of TR does not necessarily indicate the possibility of an indirect action of thyroid hormone. Several studies have suggested the presence of a non-classical thyroid hormone signaling pathway, which is non-genomic and does not require thyroid hormone interaction with the TR (59). In addition, *kiss2* gene could also be influenced by estrogen feedback via thyroid hormone action on the hypothalamic-pituitary-gonadal axis (60). Our recent report in goldfish showed presence of ERs in *kiss1* and *kiss2* neurons as well as activation of *kiss1* and *kiss2* gene promoters by estrogen (61). Currently we have no direct evidence of steroid sensitivity of *kiss2* gene in the tilapia, but our promoter analysis showed the presence of two possible ER response elements in the upstream of tilapia *kiss2* gene. Therefore, tilapia *kiss2* gene could also be influenced by estrogen in the male tilapia.

It is well known that TRH and thyroid-stimulating hormone (TSH) genes are regulated by thyroid hormone in mammals via negative feedback mechanism (62). However, in the few fish species studied, both T_4_ and T_3_ have a negative feedback effect on TSH secretion by the pituitary (63). Furthermore, it is still unknown whether T_4_ or T_3_ influences hypothalamic release of TRH in teleosts (60). In the present study, we failed to see any change in TRH mRNA expression by thyroid hormone manipulation. Similar observation has been reported in Senegalese sole that hormonal treatments using thiourea and T_4_ showed no regulation at transcriptional levels of TRH by thyroid hormones (64) and they suggested that TRH could not participate in the hypothalamic-pituitary-thyroid axis in teleosts. This is further supported by other studies that failed to demonstrate an induction of TSH or T_4_ release after TRH treatments in fish (65, 66). Therefore, in the tilapia, TRH could be insensitive to thyroid hormone levels. In addition, in mammals, not all TRH expressing neurons are T_3_ responsive (67). Therefore, it is also possible that current treatment protocol (dose and duration) in this study was not sufficient enough to alter TRH mRNA levels. We noted large variation in *gnrh3* mRNA expression in the controls in the two experiments. Such variations in *gnrh3* mRNA levels have previously been reported in teleosts (68, 69), which could be due to different social and reproductive states of fish (70–72).

In summary, we cloned *kiss2* gene in the Nile tilapia. The *kiss2* mRNA was expressed in the central and peripheral tissues. DIG-*in situ* hybridization showed *kiss2* mRNA containing cells in the nRL. Thyroid hormone (T_3_) treatment significantly increased *kiss2* and *gnrh1* mRNA levels, while those genes were suppressed under hypothyroid condition with MMI treatment. Presence of TR mRNA types in GnRH1 neurons and their absence in Kiss2 neurons suggest that GnRH1 may be directly regulated through thyroid hormone, while the regulation of Kiss2 by T_3_ is more likely to be indirect.

## Authors Contribution

Satoshi Ogawa and Ishwar S. Parhar designed the study; Satoshi Ogawa performed *in silico* gene sequence analysis, hormone treatments, data analysis, and wrote the manuscript; Kai We Ng performed cloning and real-time PCR; Xiaoyu Xue, Shuisheng Li, Berta Levavi-Sivan, Haoran Lin, Xiaochun Liu performed cloning, sequence analysis, RT-PCR; Priveena Nair Ramadasan performed *in situ* hybridization; Mageswary Sivalingam performed double-immunofluorescence and laser capture microdissection; Ishwar S. Parhar edited the manuscript, all authors approved and commented on the manuscript.

## Conflict of Interest Statement

The authors declare that the research was conducted in the absence of any commercial or financial relationships that could be construed as a potential conflict of interest.

## Footnotes

^1^http://www.cbs.dtu.dk/services/SignalP/

^2^http://asia.ensembl.org/index.html

^3^http://www.cbil.upenn.edu/cgi-bin/tess/tess

^4^http://www.cbrc.jp/research/db/TFSEARCH.html

^5^http://www-bimas.cit.nih.gov/molbio/signal/
